# Long-term education and employment outcomes in pediatric-onset systemic lupus erythematosus

**DOI:** 10.1186/1546-0096-10-S1-A105

**Published:** 2012-07-13

**Authors:** Erica F Lawson, Aimee O Hersh, Laura Trupin, Emily Von Scheven, Edward H Yelin

**Affiliations:** 1 University of California San Francisco, San Francisco, CA, USA; 2 University of Utah, Salt Lake City, UT, USA

## Purpose

Little is known about the long-term functional outcome of adults with pediatric-onset systemic lupus erythematosus (pSLE). We aim to describe education and employment outcomes in this population, and to compare subjects with pSLE to subjects with adult-onset SLE (aSLE) utilizing the UCSF Lupus Outcomes Study (LOS).

## Methods

Data derive from the 2007 cycle of the LOS, an annual longitudinal telephone survey of diverse English-speaking subjects with confirmed SLE. Only subjects of working age (22-50 years) were included in the analysis (N=326). Subjects were classified as juvenile-onset if age at diagnosis was < 18 years (N=70). Outcome variables included completion of a bachelor’s degree and current employment status. We used logistic regression to compare pSLE and aSLE with and without adjustment for other characteristics that could affect educational attainment and employment outcomes. Age, gender, ethnicity and the presence of renal disease were included as predictors of educational attainment. Predictors of employment included age, gender, ethnicity, renal disease, completion of a bachelor’s degree, self-reported disease activity according to the Systemic Lupus Activity Questionnaire (SLAQ) score, and physical disability according to the SF-36 Scale of Physical Function (SF36-PF).

## Results

Mean age of subjects was 39.2 years (SD 7.4), and 93% were female. Ethnicities included Caucasian (63%), Hispanic (9%), African American (8%), Asian (14%) and other (8%). Subjects with pSLE were more likely to be ethnic minorities (p=0.02). Mean age at diagnosis was 14 (SD 2.4) for subjects with pSLE and 28 (SD 6.5) for subjects with aSLE. Mean disease duration was 18 years (SD 7.4) for subjects with pSLE and 13 years (SD 5.3) for subjects with aSLE. Mean SLAQ score at the time of the survey was 11.2 for all subjects (SD 7.9). More subjects with pSLE had successfully completed a bachelor’s degree as compared to subjects with aSLE (49% vs. 44%), but this difference was not statistically significant. pSLE was significantly associated with lower employment rates: 51% of subjects with pSLE were currently employed, as compared to 62% of adult subjects. In multivariate logistic regression analysis, the odds of working among subjects with pSLE was less than half the odds of working for subjects with aSLE.

**Table 1:** Regression-adjusted ORs for having completed a bachelor's degree and being employed among subjects age 22-50 with SLE*

Variable	OR for having a bachelor's degree (95% CI)†	OR for being employed (95% CI)
Bachelor's degree completed		
Pediatric-onset SLE	0.89 (0.46-1.74)	0.42 (0.20-0.87)
Age	0.96 (0.92-1.00)	0.98 (0.94-1.02)
Female	0.97 (0.40-2.37)	0.62 (0.22-1.75)
Ethnicity		
Caucasian	referent	referent
Latino	0.24 (0.09-0.64)	2.21 (0.87-5.60)
African American	0.29 (0.11-0.77)	0.52 (0.20-1.38)
Asian	1.7 (0.82-3.5)	0.71 (0.33-1.54)
Other	0.55 (0.23-1.31)	0.68 (0.27-1.68)
Renal disorder	1.48 (0.89-2.49)	0.82 (0.47-1.44)
SLAQ		1.00 (0.96-1.04)
SF36-PF		1.06 (1.03-1.09)
Education¶		2.51 (1.46-4.32)

*OR = odds ratio; 95% CI = 95% confidence interval; SLAQ = Systemic Lupus Activity Questionnaire; SF36-PF = SF-36 Scale of Physical Functioning

†OR from logistic regression, adjusted for variables shown

¶Completion of bachelor's degree or higher.

## Conclusion

While subjects with pSLE are just as likely as those with aSLE to complete college education, onset of lupus in childhood significantly increases the risk of not working in adulthood, even when controlling for disease activity. Exploring reasons for low rates of employment and providing vocational support may be important to maximize long-term functional outcomes in patients with pSLE.

## Disclosure

Erica F. Lawson: None; Aimee O. Hersh: None; Laura Trupin: None; Emily Von Scheven: None; Edward H. Yelin: None.

